# Diet Quality and Consumption of Healthy and Unhealthy Foods Measured via the Global Diet Quality Score in Relation to Cardiometabolic Outcomes in Apparently Healthy Adults from the Mediterranean Region: The ATTICA Epidemiological Cohort Study (2002–2022)

**DOI:** 10.3390/nu15204428

**Published:** 2023-10-18

**Authors:** Evangelia Damigou, Matina Kouvari, Christina Chrysohoou, Fotios Barkas, Evrydiki Kravvariti, Dimitrios Dalmyras, Amalia D. Koutsogianni, Costas Tsioufis, Christos Pitsavos, Evangelos Liberopoulos, Petros P. Sfikakis, Demosthenes Panagiotakos

**Affiliations:** 1 Department of Nutrition and Dietetics, School of Health Sciences and Education, Harokopio University, 17676 Athens, Greece; 2 First Cardiology Clinic, Medical School, National and Kapodistrian University of Athens, Hippokration Hospital, 15772 Athens, Greece; 3 Department of Internal Medicine, Medical School, University of Ioannina, 45500 Ioannina, Greece; 4 First Department of Propaedeutic Internal Medicine, Medical School, National and Kapodistrian University of Athens, Laiko General Hospital, 15772 Athens, Greece

**Keywords:** Global Diet Quality Score (GDQS), diet quality, healthy foods, unhealthy foods, cardiometabolic outcomes, diabetes, cardiovascular disease

## Abstract

The Global Diet Quality Score (GDQS) is a novel food-based score that assesses both nutrient adequacy and chronic disease risk, by evaluating healthy (GDQS+) and unhealthy foods (GDQS−). The aim of this study was to evaluate the association among GDQS, GDQS+, and GDQS− against the 20-year risk of cardiometabolic outcomes in a Mediterranean population. The sample was *n* = 2169 initially free of cardiovascular disease (CVD) participants of the ATTICA study (2002–2022) that participated in the 20-year follow-up. The incidence of CVD, hypertension, hypercholesterolemia, and type 2 diabetes mellitus (T2DM) was defined according to WHO-ICD-10 criteria. The GDQS was computed based on previously published instructions. In multivariate analyses, a higher diet quality, per 1/49 of the GDQS, was associated with an 8% [95% Confidence Interval—CI: 6–9%] and 2% [95% CI: 1–3%] lower CVD and T2DM risk, respectively. A higher consumption of healthy foods, per 1/32 of GDQS+, was associated with a 9% [95% CI: 7–11%] and 2% [95% CI: 1–3%] lower CVD and T2DM risk, respectively. Contrarily, a lower consumption of unhealthy foods (GDQS−) was not associated with cardiometabolic events in the adjusted models (all *p* value< 0.05). In clinical practice or future public health actions to ameliorate dietary habits and prevent CVD and T2DM, more attention should be focused on healthy foods that should be included in our diets.

## 1. Introduction

The associations between the nutrition and development of chronic diseases are well studied and established, but still of interest, as new controversial topics arise in research [1,2,3]. A robust analysis from the Global Burden of Disease study found that 11 million deaths and 255 million disability-adjusted life years (DALYs) lost were due to dietary risks that led to chronic diseases, of which approximately 91% of deaths (i.e., 10 million) and 81% of DALYs (i.e., 207 million) were due to cardiovascular disease (CVD), followed by cancers and type 2 diabetes mellitus (T2DM) [4,5]. The burden from different CVD risk factors (in number of DALYs) usually overlaps and, based on the Global Atlas of Cardiovascular Disease, high systolic blood pressure (SBP), dietary risks, high total cholesterol, body mass index (BMI), and fasting blood glucose are among the most important CVD risk factors [6]. Moreover, a global study of the Global Cardiovascular Risk Consortium with *n* = 1,518,028 participants found that five modifiable risk factors (SBP, nonhigh-density lipoprotein cholesterol (non-HDL), BMI, diabetes, and current smoking) are responsible for 57.2% of incident CVD cases among women and 52.6% among men [7].

Concerning dietary habits, approximately 60% of chronic disease burden due to dietary risks is attributed to a high sodium intake and a low intake of whole grains and fruits [5]. However, overall dietary habits have been proposed by the American Heart Association as more important than specific foods for health outcomes [8]. Moreover, dietary patterns, such as the Mediterranean or Dietary Approaches to Stop Hypertension (DASH) diets, have been proven to be beneficial for chronic disease prevention and management [9,10]. Additionally, current lifestyles in low- and middle-income countries are affected by the double burden of malnutrition, as under- and overnutrition often coexist [11]. Scores to measure diet quality at a population level are of major importance for monitoring dietary habits and taking measures to ameliorate them.

The Global Diet Quality Score (GDQS) is a novel food-based score that comprehensively evaluates diet quality in terms of both nutrient adequacy and risk of chronic noncommunicable diseases, in contrast to most dietary scores that only evaluate one of these two components of diet quality [12]. The GDQS sums the total consumption of health-positive foods, hereinafter reported as healthy (i.e., GDQS+, higher values signify a higher consumption of these foods), as well as health-negative foods, hereinafter reported as unhealthy and unhealthy in increased amounts (i.e., GDQS−, higher scores indicate a lower consumption of these foods) [12].

The GDQS has been developed and validated for diverse populations, from both low- and high-income countries [12,13,14,15,16,17,18,19,20,21]; hence, it can be used to assess and compare different populations’ diets. Notwithstanding, due to the novelty of the score, it has been mainly evaluated in American or Asian populations; thus, its potential predictive ability of chronic noncommunicable diseases needs further research in European populations. Thus, the aim of the present study was to assess the GDQS and its submetrics (i.e., GDQS+ and GDQS−) in relation to the 20-year incidence of fatal/nonfatal CVD events and other cardiometabolic outcomes (i.e., T2DM, hypertension, and hypercholesterolemia) in apparently healthy adults from the Mediterranean region.

## 2. Materials and Methods

### 2.1. Study Design

The ATTICA study is a prospective cohort study in a sample of Greek adults with multiple follow-up examinations (i.e., baseline at 2001/2002 and a 5-, 10-, and 20-year follow-up in 2006, 2012, and 2022, respectively), which was carried out in accordance with the Declaration of Helsinki (1989) of the World Medical Association and was approved by the Institutional Ethics Committee of Athens Medical School (#017/1.5.2001). The primary aims of the study were to record the distribution of several sociodemographic, clinical, biochemical, lifestyle, and psychological parameters at various time points and to explore the associations among the aforementioned factors, their trajectories, and long-term CVD risk.

### 2.2. Setting and Participants

Participants from the province of Attica, Greece (78% urban municipalities, including Athens the capital city of Greece) were recruited. The initial sample consisted of 3042 individuals out of 4056 who were randomly invited to participate (May 2001–August 2002). Sampling was random and stratified by sex, age group, and region. Details about the study have already been published [22,23]. Of the 3042 original participants, 2169 who were found at the 20-year follow-up (2022) agreed to participate (71% participation rate). Of those lost to follow-up (*n* = 873), 771 were not found because of changed, missing, or incorrect addresses and telephone numbers and 102 because they refused to be re-screened.

### 2.3. Variables and Measurements at Baseline Examination

#### 2.3.1. Sociodemographic Parameters

Age and sex were recorded. Education status was measured as years attending school and/or college/university.

#### 2.3.2. Clinical, Biochemical, and Anthropometric Parameters

Serum lipids, blood glucose, SBP and diastolic blood pressure (DBP), waist circumference, body weight, and height were measured according to standard procedures [22]. BMI was calculated as weight/height^2^. Hypertension was defined as SBP ≥ 140 mmHg or DBP ≥ 90 mmHg (or reception of medication); hypercholesterolemia as total cholesterol >200 mg/dL (or medication); and T2DM as fasting blood glucose ≥126 mg/dL (or medication). Family history of CVD was also assessed.

#### 2.3.3. Lifestyle Habits

The Short-Form International Physical Activity Questionnaire (IPAQ) was used to evaluate physical activity levels [24]. Smoking habits during 2002–2012 were categorized into 4 different trajectories; those who never smoked during the whole 10-year period (from 2002 to 2012), those who started smoking during follow-up (2012), those who stopped smoking during follow-up (2012), and those who always smoked (2002–2012). Additionally, pack-years of cigarette smoking were calculated for each participant by multiplying smoking duration (in years) with the number of packs per day (assuming 20 cigarettes in a pack).

#### 2.3.4. Dietary Habits

At baseline examination, dietary intake was evaluated through the EPIC-Greek questionnaire, a 156-item semiquantitative food frequency questionnaire (FFQ), kindly provided by the inventors [25]. The EPIC-Greek questionnaire is validated for the Greek population and was completed with the help of trained dietitians. Participants were asked to report the average intake of each included food item during the last 12 months and to help them define the portions consumed; photographs were used. Thus, habitual intake of individual foods and food groups was recorded. Furthermore, Global Diet Quality Score (GDQS) (range: 0–49), GDQS+ (range: 0–32), and GDQS− (range: 0–17) were computed according to previously published instructions [12]. Briefly, sixteen healthy food groups (i.e., GDQS+, higher scores indicate higher consumption of healthy foods) and seven unhealthy food groups and two food groups that are unhealthy in excessive amounts (i.e., GDQS−, higher scores indicate lower consumption of unhealthy foods), comprise the GDQS, which assesses nutrient adequacy as well as risk of chronic disease [12]. Global Diet Quality Score was also used as a categorical variable; even though there is a proposed grouping method based on specific thresholds (i.e., GDQS < 15: high risk, 15–23: moderate risk, and ≥ 23: low risk), these values might be subjective; thus, in this study, comparisons were made between the sample-related tertiles of baseline GDQS. No survey scores were applied as the sampling was already stratified according to the general population.

Moreover, at all examinations (i.e., baseline and 5-, 10-, and 20-year follow-up), adherence to the Mediterranean diet was assessed through the MedDietScore [26]. For the current study, adherence to the Mediterranean diet was considered high or low based on the median value (i.e., 27/55) both at the baseline examination and the 10-year examination. Afterwards, and based on these two time points, four Mediterranean diet trajectories were formed: (i) those who were always away from the Mediterranean diet (i.e., they had a median MedDietScore value at 2002 and at 2012: <27), (ii) those who went from away (median MedDietScore at 2002: <27) to close (median MedDietScore at 2012: ≥27) to the Mediterranean diet, (iii) those who went from close (median MedDietScore score at baseline, 2002: ≥27) to away (median MedDietScore at 2012: <27) from the Mediterranean diet, and iv) those who were always close to the Mediterranean diet (median MedDietScore at 2002 and 2012: ≥27).

### 2.4. Variables and Measurements at Follow-Up Examinations

The 5-, 10-, and 20-year follow-ups included information on vital status (death from any cause), development of hypercholesterolemia, hypertension, T2DM, and fatal or nonfatal CVD. All outcomes were defined based on International Classification of Diseases (ICD)-10th version. For CVD, the ICD codes used included WHO-ICD coding 410–414.9, 427.2, and 427.6 (for acute myocardial infarction, unstable angina, or other identified forms of ischemia, respectively); WHO-ICD coding 400.0–404.9, 427.0–427.5, and 427.9 (for heart failure of different types and chronic arrhythmias); and WHO-ICD coding 430–438 (for stroke). Moreover, for the other outcomes, the ICD-10 codes used were WHO-ICD coding E11.9 for T2DM, WHO-ICD coding I10 for hypertension, and WHO-ICD coding E78.00 for hypercholesterolemia. During the 20-year follow-up, the study’s investigators (i.e., physicians) examined all participants found and ascertained all clinical outcomes of the study. Moreover, participants’ medical records were also evaluated.

### 2.5. Sample Size

**Figure 1:**
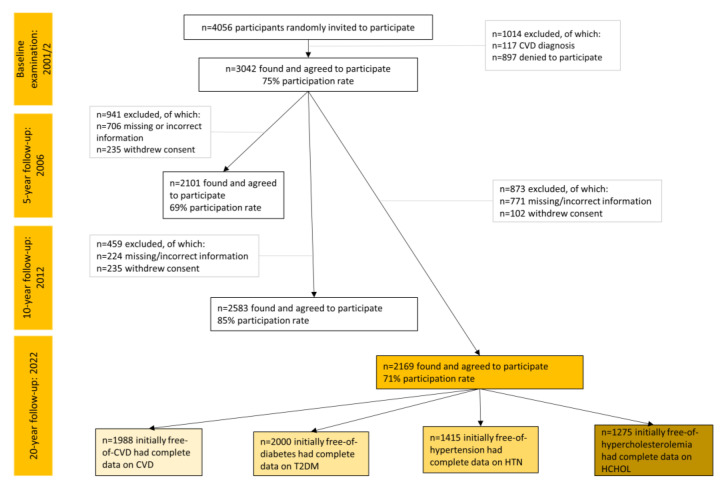
Flow chart of the ATTICA study participants during 2002–2022 (total sample *n* = 2169).

During the 20-year follow-up, in total, *n* = 2169 participants initially free of CVD were found. The age–sex distribution of this working sample and the baseline were similar (*p* > 0.80). For the different outcomes, different subsamples were used, after excluding those who were afflicted by each disease during baseline. Data for CVD were available from *n* = 1988, for T2DM from *n* = 2000, for hypertension from *n* = 1415, and for hypercholesterolemia from *n* = 1277 participants (Figure 1). Based on post hoc power calculation, the final analyzed samples were found sufficient to obtain a statistical power of >80% to detect significant between-group differences in the hazard ratios or odds ratios of CVD, T2DM, hypertension, and hypercholesterolemia (>10%), allowing for a type I error rate of 0.05.

### 2.6. Statistical Analysis

Continuous variables are presented as mean values ± standard deviation and categorical variables as frequencies. Associations between categorical variables were tested using the chi-squared test. Comparisons of mean values of normally distributed variables between the GDQS tertiles were performed using one-way analysis of variance (ANOVA), after controlling for equality of variances using Levene’s test. Continuous variables were tested for normality through P–P plots. For some continuous variables that were not normally distributed (i.e., MedDietScore, GDQS, GDQS+, GDQS−, pack-years of cigarette smoking, years in school, and food group intake) the Mann–Whitney nonparametric test was applied to evaluate the differences in the distributions of the skewed variables among those in different GDQS tertiles. Hazard ratios (HRs) and their corresponding 95% confidence intervals (95% CIs) for GDQS in relation to CVD within the 20-year period were evaluated through multivariable Cox regression analysis. Time to CVD event was recorded on annual basis. For the other outcomes, it was not possible to know the exact date to event; hence, binary logistic regression analysis was used (results are presented as Odds Ratios and their corresponding 95% CIs). Participants with missing values were excluded from the analysis. For the statistical analyses, STATA version 17 (STATA Corp, College Station, TX, USA) was used.

## 3. Results

### 3.1. Baseline Sample Characteristics

The median (interquartile range—IQR) baseline GDQS, GDQS+, and GDQS− was 37 (13), 31 (17), and 6 (0), respectively, for the total population (*n* = 2169). Median food group consumption for the total sample is presented in Table 1.

Participants’ sociodemographic, clinical, anthropometric, and lifestyle characteristics ranked from the lowest to highest GDQS tertiles are presented in Table 2. As it can be seen, differences were observed for most of these characteristics among the different groups of the GDQS tertiles.

### 3.2. Baseline Diet Quality Measured by GDQS and Its Submetrics in Relation to Long-Term Trajectories of Mediterranean Diet Adherence

Table 3 shows the mean values of the GDQS and its submetrics i.e., GDQS+ and GDQS−, in relation to the long-term trajectories of adherence to the Mediterranean diet. Of note, those who went from away (2002) to close (2012) to the Mediterranean diet, compared to those who were always away (2002–2012), had higher scores for the GDQS and GDQS− but similar scores for GDQS+.

### 3.3. Incidence of Cardiometabolic Outcomes by GDQS Tertiles

Thirty-six percent of the participants (i.e., *n* = 718 new cases) developed CVD; moreover, 54.4% developed hypercholesterolemia (*n* = 694 new cases), 22.2% developed hypertension (*n* = 314 new cases), and 26.3% developed T2DM (*n* = 526 new cases) during the 20-year follow-up.

An unadjusted analysis to evaluate the relationship between the GDQS (as a categorical variable, i.e., tertiles) and the onset of cardiometabolic outcomes during the 20-year period was performed, and the results are shown in Table 4. Those in the first tertile of the GDQS had the highest percentages of all cardiometabolic outcomes studied (i.e., fatal/nonfatal CVD events, hypercholesterolemia, hypertension, and T2DM) (all *p* < 0.05). Differences between those in the third vs. second tertiles were observed only for hypercholesterolemia and hypertension (both *p*-values < 0.05).

### 3.4. Diet Quality and Incidence of Cardiometabolic Outcomes

The results from the multiadjusted Cox proportional hazard models for CVD and binary logistic regression analysis for the other outcomes are presented in Table 5. Protective associations were observed only for CVD and T2DM. Specifically, higher GDQS, GDQS+, and GDQS tertiles (vs. tertile 1) were associated with a lower risk of incident CVD or T2DM. Concerning CVD, all associations remained significant in the fully adjusted model, while regarding T2DM, only a higher GDQS and GDQS+ were still associated with a lower risk (Table 5). In multiadjusted analyses, GDQS− was not associated with cardiometabolic events. Of note, in these analyses, we adjusted for the MedDietScore (another dietary score to assess adherence to the Mediterranean diet and thus diet quality); multicollinearity between the MedDietScore and the GDQS or GDQS+ or GDQS− was not detected (all Variance Inflation Factors—VIFs < 2); therefore, the MedDietScore remained in the models.

## 4. Discussion

In this work, we estimated diet quality, consumption of healthy, and unhealthy foods as evaluated by the GDQS, GDQS+, and GDQS−, respectively, as well as their association with a 20-year risk of cardiometabolic outcomes (i.e., CVD, T2DM, hypertension, and hypercholesterolemia) in a Mediterranean population of apparently healthy adults. We found that a higher diet quality (i.e., higher GDQS and/or GDQS tertiles) and a higher consumption of healthy foods (higher GDQS+) were inversely associated with a reduced risk of 20-year incident fatal/nonfatal CVD and T2DM, independent of long-term adherence to a Mediterranean diet and other potential confounders. We also found that a lower consumption of unhealthy foods (higher GDQS−) was not associated with the incidence of cardiometabolic outcomes. Concerning hypertension and hypercholesterolemia risk, no association was found.

It should be noted that the GDQS could be computed only at baseline; therefore, the consumption of healthy and unhealthy foods, and by extent diet quality, might have changed during the long follow-up. For that reason, we also assessed the long-term adherence to the Mediterranean diet of our working sample in relation to the baseline GDQS (Table 3). Of interest, we observed that those who ameliorated their dietary habits during the 10-year follow-up and went closer to a Mediterranean type diet, compared to those who were always away, had a slightly better baseline diet quality due to a lower consumption of unhealthy foods. Even though in our previous publications, moving closer to the Mediterranean diet did not seem to offer protection against CVD [23], we observed here that even if a lower consumption of unhealthy foods does not protect against cardiometabolic outcomes, it should be encouraged too, as it might be helpful in ameliorating dietary habits in the long term.

Although the GDQS was originally developed for nonpregnant/nonlactating women of reproductive age to assess nutritional status [12], associations have also been revealed regarding the GDQS and metabolic factors related to a variety of health conditions and disorders. In particular, an inverse relationship has been observed between the GDQS and metabolic syndrome [16]. The GDQS has been inversely associated with cardiometabolic clinical markers, such as BMI, waist circumference, total and LDL cholesterol, as well as serum ferritin levels [19]. Moreover, inverse associations have been observed among the GDQS, weight gain, and waist circumference in both men and women [15,17]. In the study by Birk et al., the GDQS as a measure of diet quality was shown to be a useful tool to identify individuals at risk of diabetes [20]. However, the relationship between the GDQS and cardiometabolic risk has not been well established and understood, as previous studies were mainly of cross-sectional design or lacking statistical power.

In our study, studying a large, population-based sample of men and women who were followed up for two decades, protective associations of the GDQS were observed for CVD and T2DM outcomes, but not other cardiometabolic outcomes, i.e., hypertension and hypercholesterolemia. To the best of our knowledge, this is the first study to assess the relationship between the GDQS and hypertension or hypercholesterolemia risk; some studies have previously shown associations with surrogate markers, i.e., blood pressure and lipid levels, or with the presence of metabolic syndrome, but not with these hard endpoints [16,19]. It is difficult to explain the lack of association observed here; however, it could be hypothesized that the relationship between the GDQS with metabolic syndrome was mainly led by the other components of the metabolic syndrome, i.e., insulin resistance and/or diabetes (as supported by our results) or the presence of central obesity (which was evaluated in this manuscript). Additionally, it could be suggested that overall diet quality may play a more prominent role for CVD and T2DM development, whereas specific nutrients or food choices might be more important for the other cardiometabolic conditions. For instance, limiting sodium intake could be more important for hypertension, while limiting saturated fat intake could be more important for hypercholesterolemia. Therefore, further research should evaluate the ability of the GDQS as a specific risk assessment tool for nutrition-related chronic diseases.

The protective actions of a high diet quality and high consumption of healthy foods on CVD and T2DM that we observed could be attributed to specific food groups. Among healthy foods, the consumption of fruits, vegetables, nuts, wholegrains, and fish rich in omega-3 fatty acids has been related to lower risks of CVD, cardiometabolic factors, cancer, and all-cause mortality [27,28,29,30,31,32,33]. Green leafy vegetables particularly have been associated with a reduced risk for CVD, although the same was not found for T2DM, especially in Asian populations [34]. However, overall dietary patterns, rather than specific foods, might be more important for T2DM [35,36,37]; it has been observed that various dietary patterns (i.e., a Mediterranean diet measured by the alternate Mediterranean diet index (aMED), a plant-based diet measured by the healthful Plant-based Diet Index (hPDI), and a healthy diet measured by the Healthy Eating Index (HEI)) were inversely associated with 624 known metabolites, which are associated with an increased risk of insulin resistance and diabetes [36]. On the contrary, consuming processed foods, such as processed meat or ultraprocessed foods, has been related to worse cardiometabolic profiles and a higher risk for CVD [3,38,39]. Increased sodium intake, due to processing or table use, has been related to CVD risk [4], although J-shape associations have also been observed [40,41]; for instance, a lower sodium intake has been recently shown to be related to both accelerated atheromatosis and lower arterial stiffness [42]. Dietary sugar and sugar-sweetened beverages have also shown detrimental effects for cardiometabolic health [43,44]. Foods, such as dairy, eggs, or unprocessed meat, have shown mixed results in the literature regarding cardiometabolic health [45,46,47,48].

We have also found that a lower consumption of unhealthy foods (as indicated by higher GDQS− values) was not associated with CVD or cardiometabolic outcomes; this may suggest that healthy food consumption is more important concerning diet quality and protection against chronic diseases, at least in our study that was conducted in a Mediterranean population with a moderate-to-high adherence to the Mediterranean diet. Thus, rather than trying to eliminate unhealthy food choices, consuming higher amounts of healthy foods may exert protective effects on cardiometabolic health. In fact, thinking of unhealthy foods as “bad” and therefore completely avoiding them is a way of thought discouraged by mindful/intuitive eating practitioners [49]. Mindful eating started as an approach to tackle disordered eating as it has been associated with weight gain prevention and better psychological health [50,51,52]. Moreover, it has been shown that providing mindful eating training to patients with T2DM led to weight loss, improved dietary habits, and glycemic control, with results similar to the commonly used diabetes self-management education [53]. Nowadays, it is also supported by various organizations and nutrition guidelines as an effective way to ameliorate dietary habits [54,55,56,57]. It should be noted that unhealthy food consumption should be limited to a minimum, because as we observed, a lower consumption of unhealthy foods was associated with better dietary habits in the long-term (i.e., ameliorated adherence to the Mediterranean diet during the follow-up). However, in future public health actions, it might be beneficial for more attention and resources to be focused on healthy foods that should be included in our diets. For instance, when trying to minimize unhealthy food consumption, fiscal methods have been established by some governments, such as taxes on foods high in salt, sugars, or saturated fat, but have not shown the expected results, as these fiscal methods usually affect mainly poorer populations [58,59,60,61,62]. On the contrary, using interventions, such as providing healthy meals in schools or prescribing healthy foods in the health care systems, offers multiple benefits not only against chronic diseases, but also for the economy, as it was proposed most recently in the U.S. through the “Food is medicine” program, supported by the American Heart Association and the Rockefeller Foundation [63,64].

### Strengths and Limitations

The ATTICA study is a prospective cohort with a long follow-up period (i.e., 20 years) and multiple waves (i.e., follow-up examinations at baseline and 5, 10, and 20 years). This fact could be considered a strength as well as a limitation. Data on dietary habits were based on a food frequency questionnaire (FFQ). Although this FFQ was semiquantitative, extensive, specific, validated for the Greek population, and used by trained dietitians, some measurement error or recall bias could have existed. Furthermore, although outcome ascertainment was verified by the study’s investigators and participants’ medical records, some incident undiagnosed cases might have been missed. Moreover, the GDQS was computed based on dietary habits only at baseline; therefore, dietary habits might have changed during the 20-year period, thereby affecting the results. However, we also took into consideration the long-term adherence to the Mediterranean diet. Even though the GDQS is a tool to evaluate dietary quality from different regions and populations, our study sample consisted only of Greek adults from an urban region; thus, this fact might have affected our results and their generalizability. Finally, the GDQS has been associated with chronic disease risk as well as nutrient inadequacy; however, in this analysis, we only assessed the relationship between the GDQS and various cardiometabolic outcomes (i.e., T2DM, hypertension, and hypercholesterolemia).

## 5. Conclusions

Our study supports the usefulness of the GDQS to evaluate diet quality in terms of preventing chronic disease, especially CVD and T2DM, in a Mediterranean population. Moreover, the study findings support the fact that in clinical practice or public health actions to ameliorate dietary habits, more attention should be focused on healthy food choices that should be included in diets, and resources should be allocated to help individuals or populations to actually do so in order to change their dietary habits for the better.

## Figures and Tables

**Table 1:** Median and interquartile ranges of food group intake (in g/day), which compose the Global Diet Quality Score for the total population (*n* = 2169).

GDQS	Food Group	Median	Interquartile Range
GDQS+	Healthy		
	Citrus fruits	90	45
	Deep orange fruits	86	46
	Other fruits	130	50
	Dark green leafy vegetables	129	50
	Cruciferous vegetables	108	25
	Deep orange vegetables	88	18
	Other vegetables	138	50
	Legumes	50	25
	Deep orange tubers	36	5
	Nuts and seeds	4	2
	Whole grains	5	10
	Liquid oils	60	5
	Fish and shellfish	21	21
	Poultry and game meat	39	19
	Low-fat dairy	29	100
	Eggs	17	9
GDQS−	Unhealthy in excessive amounts		
	High-fat dairy	30	230
	Red meat	34	17
	Unhealthy		
	Processed meat	4	6
	Refined grains and baked goods	55	10
	Sweets and ice cream	12	37
	Sugar-sweetened beverages	94	141
	Juice	143	86
	White roots and tubers	34	17
	Purchased deep fried foods	26	13

**Table 2:** Baseline sociodemographic, clinical, anthropometric, and lifestyle characteristics of participants from the ATTICA study according to Global Diet Quality Score tertiles (*n* = 2169).

Baseline Characteristics	Global Diet Quality Score (GDQS) Tertiles			
	Tertile 1	Tertile 2	Tertile 3	*p*-Value
N	804	969	396	-
Sociodemographic factors				
Age, years	50 (14)	43 (11)	40 (17)	<0.001
Men, %	58	48	38	<0.001
Education, years in school	12 (7)	12 (4)	12 (4)	0.004
Clinical factors				
History of hypercholesterolemia, %	45	41	26	<0.001
History of hypertension, %	40	25	24	<0.001
History of diabetes mellitus, %	12	4	6	<0.001
Family history of cardiovascular disease, %	37	35	36	0.309
Anthropometric factors				
Body mass index, kg/m^2^	28 (4.6)	26 (3.2)	26 (6.4)	<0.001
Waist circumference, cm	94 (15)	88 (13)	86 (19)	<0.001
Lifestyle factors				
MedDietScore, range 0–55	25 (12)	27 (1.7)	29 (6.2)	<0.001
Global Diet Quality Score, 0–49	19 (12)	37 (0)	42 (10)	<0.001
Global Diet Quality Score+, 0–32	11 (10)	31 (0)	31 (1)	<0.001
Global Diet Quality Score−, 0–17	6 (2)	6 (0)	10 (11)	<0.001
Physical activity, %yes	34	34	39	0.137
Smoking habits, %				
Never smoked (2002–2012)	40	37	39	0.002
Started smoking during follow-up (2012)	22	17	23	
Stopped smoking during follow-up (2012)	17	23	19	
Always smoked (2002–2012)	21	23	20	
Pack-years of cigarette smoking	450 (608)	340 (456)	278 (520)	<0.001

Continuous variables are presented as mean (standard deviation) or median (Interquartile Range) if normality was not met. Categorical variables as relative frequencies (percentages). *p*-values refer to differences between Global Diet Quality Score (GDQS) tertiles and categorical variables (using the chi-squared test) and continuous variables (using one-way analysis of variance-ANOVA) for normally distributed variables or the Kruskal–Wallis nonparametric test for non-normally distributed ones (i.e., MedDietScore, GDQS, GDQS+, GDQS−, pack-years of cigarette smoking, and years in school).

**Table 3:** Associations among GDQS, GDQS+, GDQS−, and Mediterranean Diet Trajectories of participants from the ATTICA study (*n* = 2169).

	Mediterranean Diet Trajectories				*p*-Value
	Always Away (2002–2012) from the Mediterranean Diet	From Away (2002) to Close (2012) to the Mediterranean Diet	From Close (2002) to Away (2012) from the Mediterranean Diet	Always Close (2002–2012) to the Mediterranean Diet	
Global Diet Quality Score, 0–49	27 (14)	30 (13) *	33 (8.5) *	35 (7) *	<0.001
Global Diet Quality Score+, 0–32	20 (12)	20 (11)	27 (8) *	28 (7) *	<0.001
Global Diet Quality Score-, 0–17	8 (5.6)	11 (5.6) *	6 (1.4) *	6 (1.1) *	<0.001

Continuous variables are presented as mean (standard deviation). *p*-Values were obtained using one-way analysis of variance. * *p* < 0.05: *p*-values from post hoc comparisons (vs. always away (2002–2012) from the Mediterranean Diet), adjusted using the Bonferroni rule.

**Table 4:** Twenty-year fatal/nonfatal cardiovascular disease incidence and intermediate cardiometabolic conditions according to Global Diet Quality Score tertiles (*n* = 2169).

20-Year Endpoint, %	Global Diet Quality Score Tertiles			*p*-Value
	Tertile 1	Tertile 2	Tertile 3	
Fatal/nonfatal cardiovascular disease event	61	22 *	21 *	<0.001
Hypercholesterolemia	80	73 *	59 *^,^ **	<0.001
Hypertension	62	43 *	35 *^,^ **	<0.001
Diabetes mellitus	38	26 *	25 *	<0.001

*p*-Values were obtained using chi-squared test. * *p* < 0.05: *p*-Values from post hoc comparisons (vs. tertile 1); ** *p* < 0.05: *p*-Values from post hoc comparisons (tertile 3 vs. tertile 2), corrected with Bonferroni rule.

**Table 5:** Sensitivity analyses to evaluate the association of Global Diet Quality Score with 20-year cardiometabolic endpoints of cardiovascular disease, hypertension, hypercholesterolemia, and type 2 diabetes mellitus.

	CVD	Hypertension	Hypercholesterolemia	Type 2 Diabetes Mellitus	
N (total), *n* (new cases)	1988, 718	1415, 314	1275, 694	2000, 526	
	HR (95% CI)	OR (95% CI)	OR (95% CI)	OR (95% CI)	**Models adjusted for**
GDQS, per 1/49	0.93 (0.92, 0.94)	0.99 (0.98, 1.00)	0.99 (0.98, 1.00)	0.98 (0.97, 0.99)	Model 1: Age, sex
GDQS+, per 1/32	0.92 (0.90, 0.93)	0.99 (0.98, 1.00)	0.98 (0.98, 1.01)	0.98 (0.97, 0.99)	
GDQS−, per 1/17	1.01 (0.96, 1.05)	1.02 (0.99, 1.05)	0.99 (0.95, 1.02)	1.00 (0.97, 1.03)	
GDQS Tertiles					
Tertile 1	Ref	Ref	Ref	Ref	
Tertile 2	0.24 (0.17, 0.33)	0.72 (0.58, 0.89)	1.01 (0.79, 1.28)	0.74 (0.59, 0.93)	
Tertile 3	0.39 (0.23, 0.67)	0.83 (0.61, 1.13)	0.82 (0.60, 1.13)	0.79 (0.57, 1.11)	
GDQS, per 1/49	0.92 (0.91, 0.94)	0.99 (0.98, 1.00)	0.99 (0.98, 1.00)	0.98 (0.97, 0.99)	Model 2: Model 1 plus body mass index, physical activity, smoking habits (2002–2012), and MedDietScore
GDQS+, per 1/32	0.91 (0.90, 0.93)	0.99 (0.98, 1.00)	0.99 (0.98, 1.01)	0.98 (0.97, 0.99)	
GDQS−, per 1/17	1.01 (0.96, 1.06)	0.99 (0.96, 1.02)	0.98 (0.94, 1.01)	1.00 (0.97, 1.03)	
GDQS Tertiles					
Tertile 1	Ref	Ref	Ref	Ref	
Tertile 2	0.23 (0.16, 0.33)	0.82 (0.66, 1.03)	1.02 (0.79, 1.31)	0.74 (0.59, 0.93)	
Tertile 3	0.31 (0.16, 0.59)	0.87 (0.60, 1.24)	0.84 (0.59, 1.19)	0.79 (0.57, 1.11)	
GDQS, per 1/49	0.92 (0.91, 0.94)	0.99 (0.98, 1.00)	0.99 (0.98, 1.00)	0.98 (0.97, 0.99)	Model 3: Model 2 plus history of (hypertension, hypercholesterolemia, and diabetes mellitus), family history of CVD, and education status
GDQS+, per 1/32	0.91 (0.89, 0.93)	0.99 (0.98, 1.00)	0.99 (0.98, 1.01)	0.98 (0.97, 0.99)	
GDQS−, per 1/17	1.02 (0.96, 1.07)	0.99 (0.95, 1.03)	0.98 (0.94, 1.02)	1.00 (0.97, 1.04)	
GDQS Tertiles					
Tertile 1	Ref	Ref	Ref	Ref	
Tertile 2	0.22 (0.14, 0.34)	0.77 (0.56, 1.12)	0.96 (0.74, 1.31)	0.78 (0.60, 1.03)	
Tertile 3	0.35 (0.17, 0.73)	0.72 (0.42, 1.26)	0.84 (0.59, 1.32)	0.78 (0.53, 1.17)	

HRs or ORs and their corresponding 95% CIs were obtained from Cox regression analysis for CVD and binary logistic regression analysis for the other outcomes, respectively. Abbreviations: CVD, Cardiovascular Disease; CI, Confidence Interval; HR, Hazard Ratio; GDQS, Global Diet Quality Score; OR, Odds Ratio.

## Data Availability

Data described in the manuscript, code book, and analytic code will be made available upon request to the corresponding author.
